# Genome Sequence of Canine Herpesvirus

**DOI:** 10.1371/journal.pone.0156015

**Published:** 2016-05-23

**Authors:** Konstantinos V. Papageorgiou, Nicolás M. Suárez, Gavin S. Wilkie, Michael McDonald, Elizabeth M. Graham, Andrew J. Davison

**Affiliations:** 1 MRC–University of Glasgow Centre for Virus Research, Glasgow, United Kingdom; 2 Division of Veterinary Pathology, Public Health and Disease Surveillance, University of Glasgow, Glasgow, United Kingdom; 3 Department of Microbiology and Infectious Diseases, Veterinary Faculty, School of Health Sciences, Aristotle University of Thessaloniki, Thessaloniki, Greece; Queen's University, CANADA

## Abstract

Canine herpesvirus is a widespread alphaherpesvirus that causes a fatal haemorrhagic disease of neonatal puppies. We have used high-throughput methods to determine the genome sequences of three viral strains (0194, V777 and V1154) isolated in the United Kingdom between 1985 and 2000. The sequences are very closely related to each other. The canine herpesvirus genome is estimated to be 125 kbp in size and consists of a unique long sequence (97.5 kbp) and a unique short sequence (7.7 kbp) that are each flanked by terminal and internal inverted repeats (38 bp and 10.0 kbp, respectively). The overall nucleotide composition is 31.6% G+C, which is the lowest among the completely sequenced alphaherpesviruses. The genome contains 76 open reading frames predicted to encode functional proteins, all of which have counterparts in other alphaherpesviruses. The availability of the sequences will facilitate future research on the diagnosis and treatment of canine herpesvirus-associated disease.

## Introduction

Canine herpesvirus (CHV; species *Canid herpesvirus 1*) was first described in 1965 as the causative agent of a fatal haemorrhagic disease of puppies [1]. It is classified in the genus *Varicellovirus* (subfamily *Alphaherpesvirinae*, family *Herpesviridae*) [2], along with related viruses such as feline herpesvirus 1 (FHV1), phocine herpesvirus 1, equine herpesvirus 1 (EHV1), pseudorabies virus (PRV) and varicella-zoster virus (VZV) [3–6].

Antigenic comparisons of various isolates indicate that CHV is monotypic [7]. The geographical seroprevalence of CHV in dogs ranges widely, and has been reported to be 88% in England, 80% in Norway, 45.8% in Belgium, 20.7% in Iran, 39.3% in the Netherlands, 39.3% in Turkey, and only 6% in the state of Washington, USA [8–14]. Although adult dogs infected with CHV do not usually show any signs, infection of susceptible puppies at 1–2 weeks of age can lead to a generalised necrotising, haemorrhagic disease [1]. Clinical signs are more likely to appear in animals that are hypothermic or immunosuppressed [15]. Like other hosts of herpesviruses, dogs become latently infected after symptomatic or asymptomatic primary infection, with CHV detectable in the trigeminal ganglia and other sites, such as the lumbosacral ganglia, tonsils and parotid salivary glands. Periodic reactivation and shedding of virus may occur in association with immunosuppression [16, 17].

The derivation of restriction endonuclease maps for CHV strain Milou indicated that the genome is 128 kbp in size and has a structure typical of varicelloviruses, described as TR_L_-U_L_-IR_L_-IR_S_-U_S_-TR_S_, in which U_L_ and U_S_ are unique long and short sequences, respectively, flanked by terminal and internal inverted repeats TR_L_/IR_L_ and TR_S_/IR_S_, respectively [5]. The sizes of U_L_, U_S_, TR_L_/IR_L_ and TR_S_/IR_S_ were estimated at 100 kbp, 7.4–8.6 kbp, 37 bp and 10.1–10.7 kbp, respectively. The same study reported an analysis of partial sequence data obtained from regions throughout U_L_, comprising about 20% of the genome, and this led to the identification of sequences homologous to 35 open reading frames (ORFs) in other alphaherpesviruses. In another study, a 10,592 bp sequence comprising U_S_ (7,678 bp) and flanking portions of TR_S_/IR_S_ in CHV strain D004 was determined [18]. In addition, shorter sequences are available from various CHV genes [19–30]. In this study, we report the genome sequences of three CHV isolates and an analysis of their genetic content.

## Materials and Methods

### Viral strains

Three CHV strains were archived as isolates recovered from diagnostic specimens at Veterinary Diagnostic Services, Small Animal Hospital, School of Veterinary Medicine, University of Glasgow. CHV strain 0194 (CHV/0194) was recovered from an unknown organ and breed in 1985, strain V777 (CHV/V777) was isolated from the lung of an 11 day-old miniature Schnauzer in 1995, and strain V1154 (CHV/V1154) originated from the kidney of a 14 day-old Dalmatian in 2000.

### DNA extraction and library preparation

The isolates were recovered in Madin-Darby canine kidney cells (American Type Culture Collection, Manassas, VA, USA) by using standard cell culture techniques, and cell-released virus was pelleted from the infected cell medium at a late stage of infection by ultracentrifugation at 145,500 *g* for 3 h. The pellet was resuspended in 200 μl 10 mM Tris-HCl (pH 8) and centrifuged at 900 *g* for 3 min to pellet cellular debris. DNA was extracted from the supernatant by using a DNeasy blood and tissue kit (Qiagen, Crawley, UK), and quantified by using a Qubit 2.0 fluorometer (Life Technologies Co., Carlsbad, CA, USA). Aliquots of DNA from CHV/0194, CHV/V777 and CHV/V1154 (0.565 μg, 0.605 μg and 0.336 μg, respectively) were sheared acoustically to an average size of 460 nucleotides (nt) in a volume of 50 μl by using a Covaris S220 sonicator (Covaris Inc., Woburn, MA, USA). Fragment size was measured by using an Agilent 2200 Tapestation (Agilent, Santa Clara, CA, USA). A KAPA library preparation kit (KAPA Biosystems, Wilmington, MA, USA) was used to prepare the sheared DNA fragments for Illumina sequencing, as described previously [31].

### DNA sequencing

A MiSeq running v3 chemistry (Illumina, San Diego, CA, USA) was used to generate 1,592,240, 1,607,590 and 1,499,502 paired-end reads of 300 nt from the CHV/0194, CHV/V777 and CHV/V1154 libraries, respectively. Data quality was assessed by using FastQC [32], and 80 nt were removed from the 3’ ends of all reads by using PrinSeq [33]. The reads for CHV/0194 and CHV/V777 were assembled *de novo* into contigs by using SPAdes 3.5.0 [34], and a template was constructed from the contigs for each strain. The reads were then aligned against the appropriate template by using Bowtie2 [35], and the alignment was visualised by using Tablet v.1.13.08.05 [36]. For CHV/0194, 444,783 reads aligned at an average coverage depth of 732 reads/nt, and for CHV/V777 these values were 161,143 and 267, respectively. The genome termini were identified from published information [5]. Since the CHV/V1154 library contained significantly fewer viral reads, *de novo* assembly resulted in a low quality template. Hence, the reads were aligned not against this sequence but against the CHV/0194 template, with 34,091 reads aligning at an average coverage depth of 41 reads/nt. Mismatches in the template were then corrected manually.

### Nucleotide sequence accession numbers

The CHV/0194, CHV/V777 and CHV/V1154 genome sequences were deposited in GenBank under accession numbers KT819633, KT819632 and KT819631, respectively. The sequence read datasets were deposited in the European Nucleotide Archive under accession numbers ERS1026451, ERS1026452 and ERS1026453, respectively, in study number PRJEB12251.

### Bioinformatic analysis

Standard programs used to analyse the sequences included the EMBOSS [37], ExPASy [38] and NCBI suites [39], as well as SignalP 4.1 [40] and Philius [41]. The locations of ORFs encoding functional proteins were predicted initially by identifying all ATG-initiated ORFs larger than 50 codons. ORFs overlapping larger ORFs for more than half their length and lacking significant amino acid sequence similarity to recognised proteins (particularly from alphaherpesviruses) were then discounted. The first ATG in each ORF was assigned as the initiation codon, except in a few instances in which the use of a subsequent ATG was supported by alignments with orthologues from other herpesviruses or by the presence of a putative signal peptide.

For phylogenetic analysis, amino acid sequences were extracted from GenBank for FHV1 (NC_013590), EHV1 (NC_001491), equine herpesvirus 4 (EHV4, NC_001844), equine herpesvirus 3 (EHV3, NC_024771), bovine herpesvirus 1 (BoHV1, NC_001847), bovine herpesvirus 5 (BoHV5, NC_005261), PRV (NC_006151), VZV (NC_001348), simian varicella virus (SVV, NC_002686) and herpes simplex virus type 1 (HSV1, NC_001806). Mega 6.06 [42] was used to compute phylogenies, using ClustalW to align sequences and an LG+G+F model with 100 bootstraps to calculate trees.

## Results

### Genome characteristics

The genome sequence obtained for CHV/0194 is 125,171 bp in size and exhibits an organization typical of that of varicelloviruses. The overall nucleotide composition is 31.6% G+C. As in other alphaherpesviruses, regardless of overall nucleotide composition, the G+C content of TR_S_/IR_S_ is higher than that of the rest of the genome, at 36.8% [6, 43]. The sizes of U_L_, U_S_, TR_L_/IR_L_ and TR_S_/IR_S_ are 97,465, 7,678, 38 and 9,976 bp, respectively. The corresponding sizes for CHV/V777 are closely similar to those of CHV/0194: total length, 124,744 bp; U_L_, 97,220 bp; U_S_, 7,678 bp; TR_L_/IR_L_, 38 bp; and IR_S_/TR_S_, 9,885 bp.

Twelve different tandem repeats of a unit longer than 10 bp were identified in the CHV/0194 genome, seven of these in U_L_ and five duplicated in TR_S_/IR_S_ (Table 1). Like the genome as a whole, several have a low G+C content. Five contain units consisting of multiples of 3 nt and encode repeated amino acid sequences in recognised proteins. The unit numbers in four repeats (36302–36608, 92993–93399, 102685–103063 and 103032–103318) were not determined directly, because the overall repeat length appeared to exceed the read length. Consequently, the numbers resulting from *de novo* assembly, which takes into account the fact that the paired-end reads were produced from DNA fragments of a measured average size, were adopted. The same twelve repeats were recognised in the CHV/V777 genome, and, although the units had the same sequences as in CHV/0194, their numbers were not necessarily the same. The numbers of units in the four repeats listed above, and that in 16274–16475, were not determined directly. The repeats in CHV/V1154 have the same unit sequences as those in the other two strains, but, since derivation of the genome sequence did not depend on *de novo* assembly, the repeats of unresolved lengths were treated as gaps and, as a result, the sequence was considered to be incomplete. Further details are available in the GenBank entries.

**Table 1:** Tandem repeats in the CHV/0194 genome.

Location (bp)	Length (bp)	Unit (bp)	Unit no.	Partial unit (bp)	Unit sequence (5’-3’)	Repeat G+C (%)
127–224	98	12	8	2	TCCCATAACCCC	58
16274–16475	202	63	3	13	TATCAACACCCGCGGAGAACAAGCTCCAGAATTCAGTCCACGGAGTCTGAGTCTAATTTTGAC	48
35395–35666	272	30	9	2	AAACAACCAACCACAGTCCAGCAACCCGCC	56
36167–36301	135	24	5	15	CCAAGAACCTCAGCGTCCCAGAGT	59
36302–36608	307	24	12	19	ACGGGGAACCCGAGGACGCCAGCG	75
62836–62985	150	58	2	34	TACTGGGATCGGGGGGTTGAGGACGCGGATGGTTCACGGCACGCCGGATCGAGCGTGA	65
92993–93399	407	72	5	47	ACTATTAGAATTAACACTCTTACGTCTAGATTGTTTCAACTCTGATGCATCTCCCAACTTCTCTGTAGAATA	34
97619–97766 125094–124947	148	34	4	12	ATAGTCCAACCCCCTTAGGCCCCGCCCACTCAAT	58
102509–102676 120204–120037	168	52	3	12	CATGTTGATCCTCCCTCTTTGTGTATCCCATTTGCGTGTGTAATTAGGCCGC	48
102685–103063 120028–119650	379	53	7	8	ATATTTAAATTGGCTGCCATGTAAACCCTCCCTCTATTACGTGTGTAATTTAC	35
103032–103318 119681–119395	287	40	7	7	CCCTCTATTACGTGTGTAATTTACATATTTAATTGAATCA	28
105507–105763 117206–116950	257	13	19	10	AAATCTATGAATG	23

The three genomes are very closely related, any one differing from each of the others by only 22 nucleotide substitutions (99.98% identical). They also differ in the numbers of units in the repeats described above or other, smaller repeats, although the units have the same sequence in each strain. This reflects a widely recognised phenomenon in herpesviruses, in which repeats may vary in length among strains or even within the same strain as a result of recombination. We identified a total of 95 sequences for CHV strains deposited in GenBank, ranging in size from 171 to 10,592 bp, and all of these are also highly similar to the corresponding CHV/0194, CHV/V777 and CHV/V1154 sequences. For example, the four largest sequences exhibit the following levels of identity to the corresponding regions of the CHV/0194 genome (not counting repeats with different unit numbers): 100% in 10,592 bp containing U_S_ and adjacent regions of TR_S_/IR_S_ (U84223; USA strain D004) [18]; 99.86% in 6,562 bp containing UL25-UL21 (AF361075; Australian strain AUS2) [25]; 99.98% in 6,323 bp containing part of U_S_ (AF361076; Australian strain AUS2) [25]; and 99.74% in 5,580 bp containing RS1 (AB012086; Japanese strain GCH-1) [27].

### Predicted protein-coding regions

**Fig 1:**
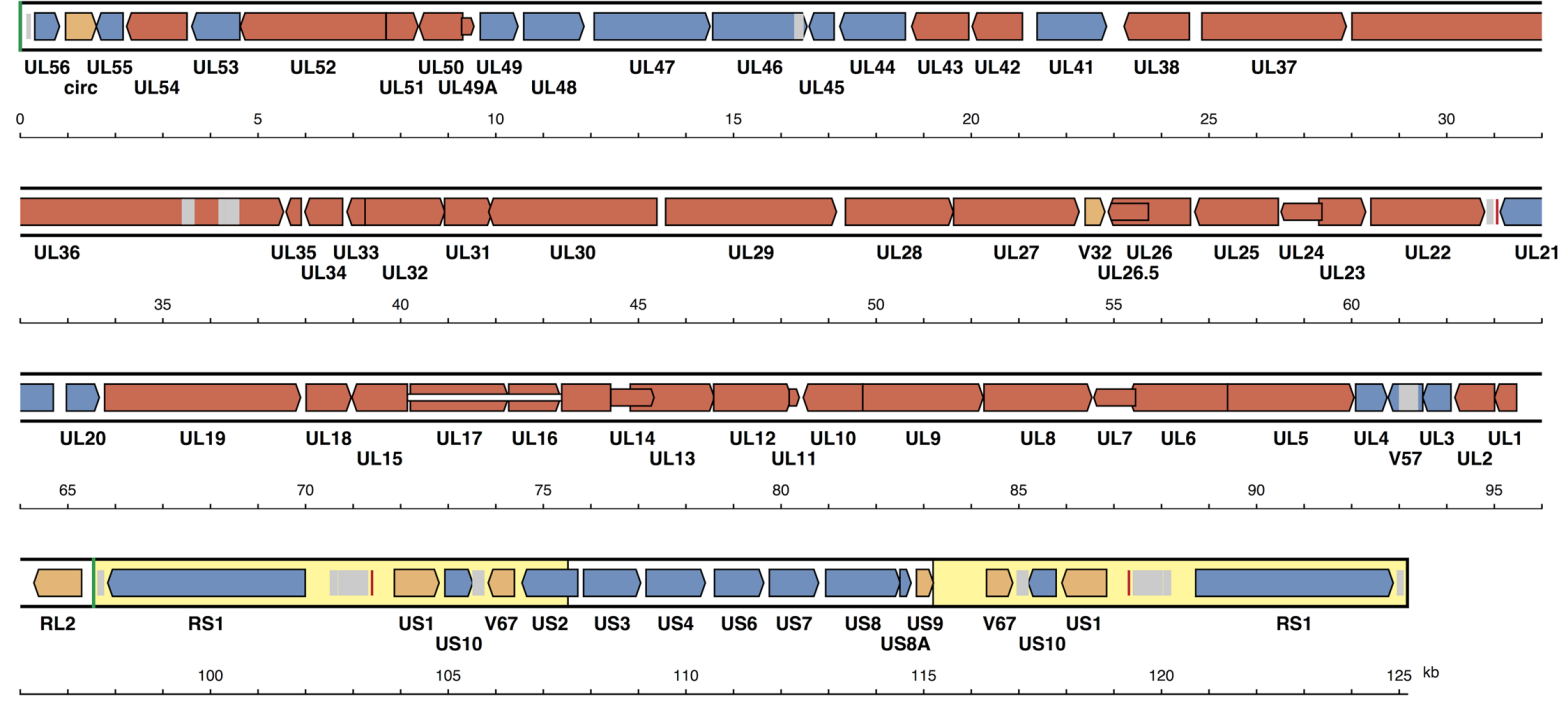
Map of the CHV/0194 genome. ORFs are shown by coloured arrows, with names below. Red shading shows ORFs that were inherited from the ancestor of family *Herpesviridae*, blue shading shows ORFs that were inherited from the ancestor of subfamily *Alphaherpesvirinae*, and orange shading shows ORFs that evolved within subfamily *Alphaherpesvirinae*. Inverted repeats TR_S_/IR_S_ are shaded yellow, and inverted repeats TR_L_/IR_L_ are shown by green vertical lines. Tandem repeats are marked by grey shading, and origins of DNA replication by vertical red lines.

The map of the CHV/0194 genome (Fig 1) contains a total of 76 ORFs predicted to encode functional proteins, one of which (UL15) is spliced. Of these ORFs, 61 are located in U_L_ and seven in U_S_, and the four mapping in TR_S_ are duplicated in IR_S_. One ORF (US2) starts in U_S_ and ends in IR_S_, and it is possible that an N-terminally truncated version of the encoded protein is expressed from a shorter ORF in TR_S_ (not included in Fig 1). Table 2 lists the properties and functions assigned to the predicted CHV proteins, based on studies of their counterparts in other alphaherpesviruses [44].

**Table 2:** Amino acid sequence identity between CHV and FHV1 genes.

Gene	Protein	Identity (%)
UL56	Membrane protein UL56	19.6
circ	Myristylated tegument protein CIRC	42.8
UL55	Nuclear protein UL55	52.9
UL54	Multifunctional expression regulator	48.0
UL53	Envelope glycoprotein K	63.7
UL52	Helicase-primase primase subunit	46.1
UL51	Tegument protein UL51	50.6
UL50	Deoxyuridine triphosphatase	43.1
UL49A	Envelope glycoprotein N	52.6
UL49	Tegument protein VP22	32.3
UL48	Transactivating tegument protein VP16	59.4
UL47	Tegument protein VP13/14	43.1
UL46	Tegument protein VP11/12	40.0
UL45	Membrane protein UL45	30.1
UL44	Envelope glycoprotein C	37.8
UL43	Envelope protein UL43	38.8
UL42	DNA polymerase processivity subunit	45.3
UL41	Tegument host shutoff protein	63.5
UL38	Capsid triplex subunit 1	55.8
UL37	Tegument protein UL37	44.3
UL36	Large tegument protein	41.8
UL35	Small capsid protein	68.5
UL34	Nuclear egress membrane protein	49.5
UL33	DNA packaging protein UL33	54.3
UL32	DNA packaging protein UL32	56.7
UL31	Nuclear egress lamina protein	58.9
UL30	DNA polymerase catalytic subunit	63.5
UL29	Single-stranded DNA-binding protein	69.4
UL28	DNA packaging terminase subunit 2	61.2
UL27	Envelope glycoprotein B	72.2
V32	Protein V32	41.4
UL26	Capsid maturation protease	48.5
UL26.5	Capsid scaffold protein	38.4
UL25	DNA packaging tegument protein UL25	61.1
UL24	Nuclear protein UL24	50.2
UL23	Thymidine kinase	42.7
UL22	Envelope glycoprotein H	40.2
UL21	Tegument protein UL21	53.6
UL20	Envelope protein UL20	60.9
UL19	Major capsid protein	75.3
UL18	Capsid triplex subunit 2	75.4
UL17	DNA packaging tegument protein UL17	51.1
UL16	Tegument protein UL16	56.3
UL15	DNA packaging terminase subunit 1	70.7
UL14	Tegument protein UL14	37.4
UL13	Tegument serine/threonine protein kinase	48.8
UL12	Deoxyribonuclease	59.3
UL11	Myristylated tegument protein	53.9
UL10	Envelope glycoprotein M	47.8
UL9	DNA replication origin-binding helicase	58.4
UL8	Helicase-primase subunit	52.6
UL7	Tegument protein UL7	52.4
UL6	Capsid portal protein	59.7
UL5	Helicase-primase helicase subunit	70.4
UL4	Nuclear protein UL4	46.9
V57	Protein V57	21.5
UL3	Nuclear protein UL3	66.2
UL2	Uracil-DNA glycosylase	54.2
UL1	Envelope glycoprotein L	39.7
RL2	Ubiquitin E3 ligase ICP0	24.3
RS1	Transcriptional regulator ICP4	44.8
US1	Regulatory protein ICP22	42.2
US10	Virion protein US10	40.8
V67	Virion protein V67	^a^
US2	Virion protein US2	^b^
US3	Serine/threonine protein kinase US3	51.2
US4	Envelope glycoprotein G	41.9
US6	Envelope glycoprotein D	40.5
US7	Envelope glycoprotein I	37.9
US8	Envelope glycoprotein E	46.8
US8A	Membrane protein US8A	18.0
US9	Membrane protein US9	30.5

^a^ V67 is absent from FHV1.

^b^ Only a short fragment of US2 is present in FHV1, perhaps indicating a deletion or assembly error that might also affect V67.

### Phylogenetic relationships

**Fig 2:**
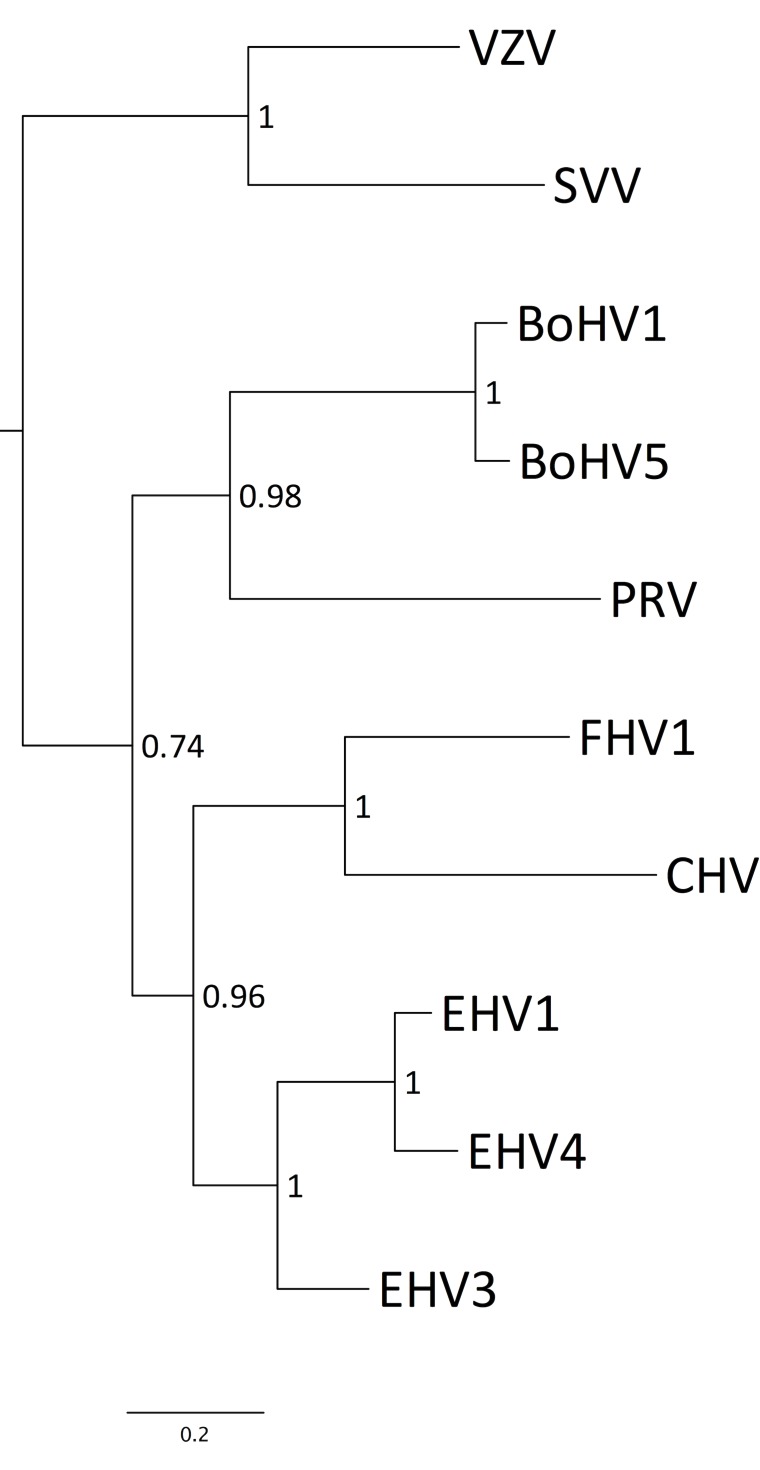
Phylogenetic tree of varicellovirus DNA polymerases. The tree was rooted on HSV1, which is a member of genus *Simplexvirus*. Bootstrap values are indicated at nodes as fractions. The scale bar indicates substitutions per amino acid residue.

Phylogenetic analysis of the DNA polymerase genes (UL30) of CHV and other completely sequenced varicelloviruses showed that CHV is most closely related to another carnivore herpesvirus (FHV1) and then to equine herpesviruses (Fig 2). Similar trees were obtained for UL19, UL15, UL9 and RS1 (data not shown). Levels of amino acid sequence identity between CHV and FHV1 range from 75.4% (UL18) to 18.0% (US8A) (Table 2). All CHV ORFs have orthologues in other varicelloviruses, although 11 are absent from some: UL56 (BoHV1 and BoHV5), circ (PRV and SVV), UL55 (BoHV1, BoHV5 and PRV), UL45 (BoHV1, BoHV5, PRV, VZV and SVV), V32 (BoHV1, BoHV5 and PRV), US10 (BoHV1, BoHV5 and PRV), V67 (FHV1, PRV, VZV and SVV), US2 (VZV and SVV), US4 (VZV and SVV), US6 (VZV and SVV) and US8A (BoHV1, BoHV5, PRV, VZV and SVV). Two ORFs (V32 and V67) have orthologues only among the varicelloviruses and not other alphaherpesviruses [44]. US8A lacks convincing sequence similarity to other alphaherpesvirus ORFs and was assigned as a positional orthologue [23].

### Origins of DNA replication

By analogy to other alphaherpesviruses, including pseudorabies virus [45], three well-defined origins of DNA replication were predicted in the CHV genome, each consisting of inverted copies of a sequence (TTCGCAC), which binds to the DNA replication origin-binding helicase encoded by UL9, separated by a partially palindromic, A+T-rich sequence. Two copies of oriS are located in TR_S_/IR_S_ between RS1 and US1, and the copy of oriL is located near the centre of U_L_, between UL22 and UL21.

## Discussion

The sizes of the CHV/0194 and CHV/V777 genomes determined from the complete sequences (125 kbp) are close to that (128 kbp) estimated by restriction endonuclease mapping of strain Milou [5]. This is at the lower end of the size range for varicelloviruses and closely similar to that of VZV [44]. The completely sequenced varicelloviruses range in nucleotide composition from 31.6 (CHV) to 74.8% G+C (BoHV5; [46]), a differential phenomenon that extends across the entire genome and has been noted previously as resulting in a high degree of codon bias [43]. Analysis of the complete genome sequences of the CHV strains thus confirmed the remarkably low G+C content observed previously [5, 19] [47]. The observation that CHV/0194, CHV/V777 and CHV/V1154 are highly similar in sequence, despite having been isolated over a 15 year period, combined with a corresponding degree of similarity to the partial sequences of many other strains, indicates that the CHV genome lacks significant diversity, which is consistent with the description of the virus as monotypic.

Two adjacent genes (UL39 and UL40) encoding the large and small subunits of ribonucleotide reductase, respectively, are conserved in other alphaherpesviruses infecting mammals, birds or reptiles, but are not present in CHV [44, 48]. Their absence from the anticipated location in the genome was noted previously from partial sequence data for strain Milou, suggesting that they had either been transferred to new locations or lost completely, or that this strain may have suffered a deletion during isolation [5]. The partial data for strain Milou and the complete data for independently isolated strains CHV/0194, CHV/V777 and CHV/V1154 confirm that these genes have indeed been lost completely during CHV evolution, and that this loss is not an artefact of viral isolation. There is precedent for a virally encoded ribonucleotide reductase not being necessary for herpesvirus pathogenesis, in that betaherpesviruses lack the small subunit, retaining only the large subunit in a form that is enzymatically inactive but nonetheless required for pathogenesis [49].

In conclusion, we have used high-throughput methods to determine the genome sequences of three CHV strains isolated in the UK over a 15 year period. Analyses of these sequences demonstrated a very high degree of similarity among strains, and provided a detailed gene map for the entire genome. The availability of the sequences will aid future research on CHV, in particular that directed at diagnosis and intervention.
